# Oral Bacteria Combined with an Intranasal Vaccine Protect from Influenza A Virus and SARS-CoV-2 Infection

**DOI:** 10.1128/mBio.01598-21

**Published:** 2021-08-17

**Authors:** Minami Nagai, Miyu Moriyama, Takeshi Ichinohe

**Affiliations:** a Division of Viral Infection, Department of Infectious Disease Control, International Research Center for Infectious Diseases, Institute of Medical Science, The University of Tokyo, Minato-ku, Tokyo, Japan; Johns Hopkins Bloomberg School of Public Health

**Keywords:** mucosal immunity, intranasal vaccine, adjuvant, SARS-CoV-2

## Abstract

The gut microbiota plays a critical role in the induction of adaptive immune responses to influenza virus infection. However, the role of nasal bacteria in the induction of the virus-specific adaptive immunity is less clear. Here, we found that disruption of nasal bacteria by intranasal application of antibiotics before influenza virus infection enhanced the virus-specific antibody response in a MyD88-dependent manner. Similarly, disruption of nasal bacteria by lysozyme enhanced antibody responses to intranasally administered influenza virus hemagglutinin (HA) vaccine in a MyD88-dependent manner, suggesting that intranasal application of antibiotics or lysozyme could release bacterial pathogen-associated molecular patterns (PAMPs) from disrupted nasal bacteria that act as mucosal adjuvants by activating the MyD88 signaling pathway. Since commensal bacteria in the nasal mucosal surface were significantly lower than those in the oral cavity, intranasal administration of HA vaccine alone was insufficient to induce the vaccine-specific antibody response. However, intranasal supplementation of cultured oral bacteria from a healthy human volunteer enhanced antibody responses to an intranasally administered HA vaccine. Finally, we demonstrated that oral bacteria combined with an intranasal vaccine protect from influenza virus and severe acute respiratory syndrome coronavirus 2 (SARS-CoV-2) infection. Our results reveal the role of nasal bacteria in the induction of the virus-specific adaptive immunity and provide clues for developing better intranasal vaccines.

## INTRODUCTION

Respiratory infectious diseases, such as influenza and coronavirus disease 2019 (COVID-19), cause substantial morbidity and mortality. Influenza A virus is responsible for annual epidemics that cause severe morbidity and mortality involving 3 to 5 million people worldwide. In addition, the constant pandemic potential of newly emerging viruses remains a serious threat to public health, the economy, and society, as illustrated by the recent COVID-19 global pandemic. Therefore, there is an urgent need to develop effective vaccines against not only seasonal influenza viruses but also severe acute respiratory syndrome coronavirus 2 (SARS-CoV-2).

Since it is difficult to predict which strain of influenza virus or coronavirus will cause a pandemic, it is advantageous to produce vaccines that induce cross-protective immunity against variants of the particular virus strain. Mucosal immunity induced by intranasal vaccination with an influenza vaccine is more effective and cross-protective against heterologous virus infection than systemic immunity induced by parenteral vaccines (1). It is believed that the virus-specific IgA in the upper respiratory tract is more cross-protective against heterologous influenza viruses than the virus-specific IgG in the serum due to its dimeric or tetrameric forms (higher avidity) and location (2, 3). Indeed, polymeric immunoglobulin receptor-knockout mice failed to secrete nasal IgA and protect against heterologous virus challenge (4). In the effort to develop effective intranasal vaccines, several adjuvants, such as cholera toxin (CT) (5), synthetic double-stranded RNA poly(I:C) (6), synthetic Toll-like receptor 4 agonist (7), zymosan (8), flagellin (9), immune stimulating complexes (ISCOMs) (10), or type I interferons (11), have been developed to enhance the vaccine-specific nasal IgA response. While the upper respiratory tract contains commensal bacteria (12, 13), intranasal administration of a split vaccine alone was insufficient to induce the vaccine-specific nasal IgA response (6, 14), suggesting that the amounts of commensal bacteria in the upper respiratory tract are insufficient to stimulate the vaccine-specific nasal IgA response.

Recently, it has become increasingly apparent that gut microbiota play a critical role not only in the induction of adaptive immune responses but also in innate antiviral immune responses to influenza virus infection (15–21). In contrast to the role of gut microbiota in antiviral resistance to influenza virus infection, it remains unclear whether nasal bacteria critically regulate the generation of influenza virus-specific adaptive immune responses after infection or intranasal vaccination. Here, we show that depletion of nasal bacteria by intranasal administration of antibiotics enhanced the virus-specific nasal IgA and serum IgG response following influenza virus infection. In addition, we found that lysozyme-induced disruption of nasal bacteria or intranasal administration of cultured oral bacteria from a healthy volunteer significantly enhanced the vaccine-specific nasal IgA and serum IgG responses in a MyD88-dependent manner. Our results reveal the role of nasal bacteria in the induction of the virus-specific adaptive immunity and provide clues for developing better intranasal vaccines.

## RESULTS

### Depletion of nasal bacteria enhanced antibody response to influenza virus infection.

**FIG 1:**
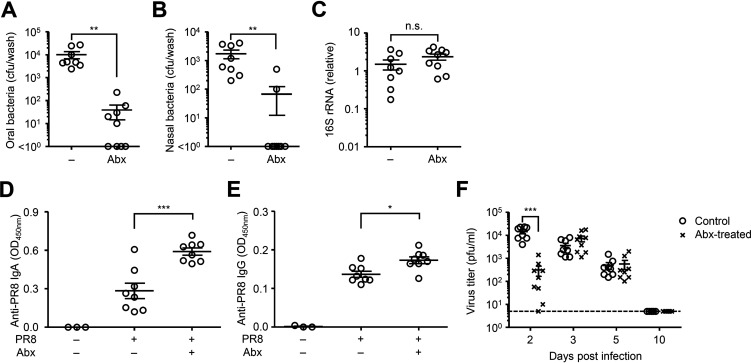
Disruption of nasal bacteria enhances the virus-specific antibody responses following influenza virus infection. (A to C) Mice were inoculated intranasally with an antibiotic cocktail (Abx) for 5 consecutive days. Two days later, tongue (A), nasal wash (B), and stool (C) were collected. Bacterial load in the tongue (A) and nasal wash (B) or relative gene copies of 16S rRNA isolated from stool pellets (C) were measured. (D to F) Mice were inoculated intranasally with an Abx for 5 consecutive days. Two days later, mice were intranasally infected with 1,000 PFU of A/PR8 virus. The nasal wash and serum were collected at 4 weeks p.i., and the virus-specific nasal IgA (D) and serum IgG titers (E) were determined by ELISA. The nasal wash was collected at indicated time points, and viral titers were determined by plaque assay. (F) Each symbol indicates values for individual mice. The data are from three independent experiments (mean ± SEM). *, *P < *0.05; **, *P < *0.01; ***, *P < *0.001; n.s., not significant (one-way ANOVA and Tukey’s test).

Gut commensal microbiota play a key role in innate and adaptive immune defense against influenza virus infection (15–17, 19, 21, 22). However, the role of nasal bacteria in the induction of mucosal immune responses following influenza virus infection remains unknown. To assess the effects of nasal bacteria in the induction of mucosal immune responses to influenza virus infection, we treated mice intranasally with an antibiotic cocktail for 5 consecutive days before influenza virus infection. This treatment resulted in a significant reduction in the numbers of culturable oral and nasal bacteria without affecting the amount of 16S rRNA in the gut (Fig. 1A to C). Antibiotic-treated mice were then infected intranasally with a mouse-adapted influenza A virus strain, A/Puerto Rico/8/1934 (PR8). Surprisingly, influenza virus-specific nasal IgA and serum IgG levels were significantly elevated in the antibiotic-treated group (Fig. 1D and E). This elevation led us to consider the possibility that depletion of commensal bacteria in the upper respiratory tract enhances influenza virus replication, resulting in enhancement of the virus-specific antibody response. However, depletion of commensal bacteria in the upper respiratory tract significantly reduced influenza virus replication at 2 days postinfection (Fig. 1F). This finding is consistent with a previous report showing that antibiotic treatment significantly reduces influenza virus replication at early time points (23). In addition, the viral replication in the upper respiratory tract became comparable between antibiotic-treated and control groups at 3 and 5 days postinfection and completely cleared the virus by 10 days postinfection (Fig. 1F). These data indicated that the levels of influenza virus replication in the upper respiratory tract are unlikely to account for the increased the virus-specific antibody response in antibiotic-treated animals.

### Lysozyme-induced disruption of nasal bacteria enhances antibody response induced by intranasal vaccination.

**FIG 2:**
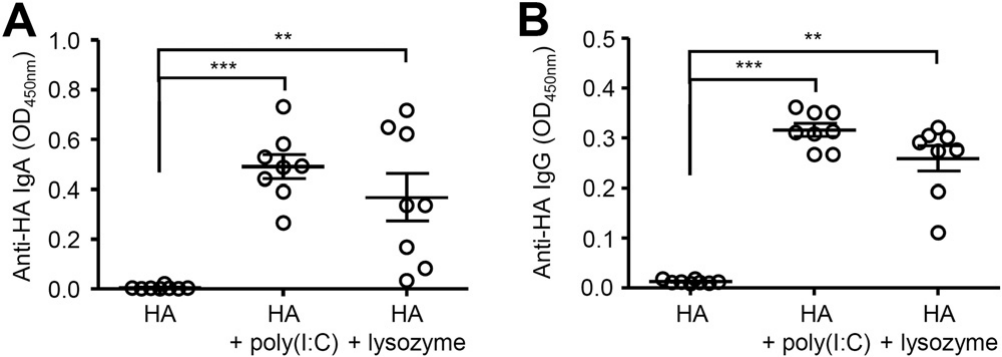
Disruption of nasal bacteria induces the HA-specific antibody responses after intranasal vaccination. (A and B) Mice were immunized intranasally with quadrivalent HA vaccine with or without poly(I:C) or lysozyme twice in a 3-week interval. Two weeks later, the nasal washes and sera were collected and the HA-specific nasal IgA and serum IgG titers were determined by ELISA. Open circles indicate values for individual mice. The data are from three independent experiments (mean ± SEM). **, *P < *0.01; ***, *P < *0.001 (one-way ANOVA and Tukey’s test).

Thus, we next examined the possibility that antibiotics induced a disruption of nasal bacterial PAMPs, which may act as adjuvants to enhance the virus-specific antibody response. To assess this possibility, we immunized mice intranasally with the influenza virus hemagglutinin (HA) protein and lysozyme to disrupt nasal bacteria. We used a poly(I:C) adjuvant as a positive control (6). Strikingly, we found that intranasal immunization with HA and lysozyme significantly enhanced the HA-specific nasal IgA and serum IgG responses (Fig. 2). While the upper respiratory tract contains commensal bacteria (12, 13), intranasal administration of the hemagglutinin (HA) vaccine alone was insufficient to induce the HA-specific antibody response (Fig. 2). Taken together, these results suggest that disruption of nasal bacteria by intranasal administration of antibiotics or lysozyme enhances antibody responses to intranasally administered vaccines.

### Intranasal supplementation of cultured oral bacteria enhances antibody response to intranasally administered vaccine.

**FIG 3:**
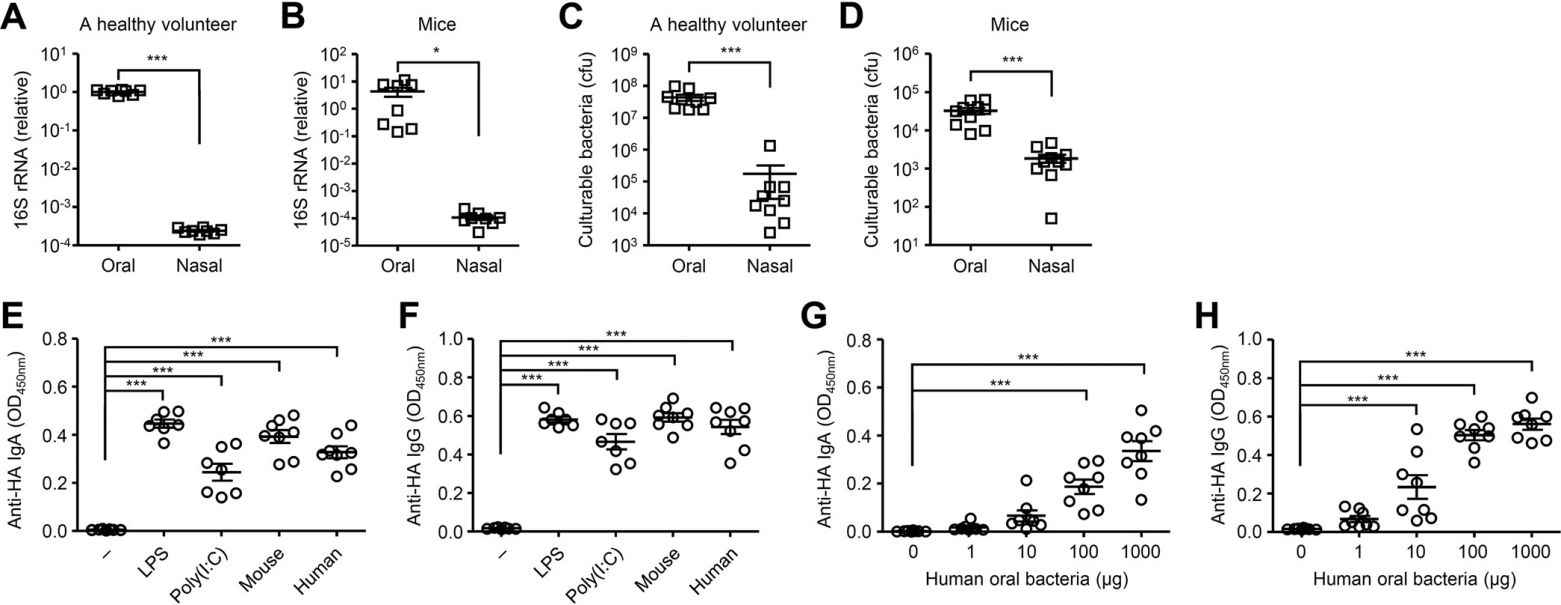
Cultured oral bacteria stimulate the HA-specific antibody responses. (A and B) Relative gene copies of 16S rRNA isolated from tongue (A) and nasal wash (B) were quantified by quantitative PCR (qPCR). (C and D) Culturable bacterial load in the tongue (C) and nasal wash (D) were measured. (E and F) Mice were immunized intranasally with quadrivalent HA vaccine with or without LPS, poly(I:C) or cultured oral bacteria from mice or a healthy volunteer twice in a 3-week interval. Two weeks later, the nasal washes and sera were collected and the HA-specific nasal IgA and serum IgG titers were determined by ELISA. (G and H) Mice were immunized intranasally with quadrivalent HA vaccine with or without indicated amounts of oral bacteria from a healthy volunteer twice in a 3-week interval. Two weeks later, the nasal washes and sera were collected and the HA-specific nasal IgA and serum IgG titers were determined by ELISA. Each symbol indicates values for individual mice. The data are from two independent experiments (mean ± SEM). *, *P < *0.05; ***, *P < *0.001 (one-way ANOVA and Tukey’s test).

**FIG 4:**
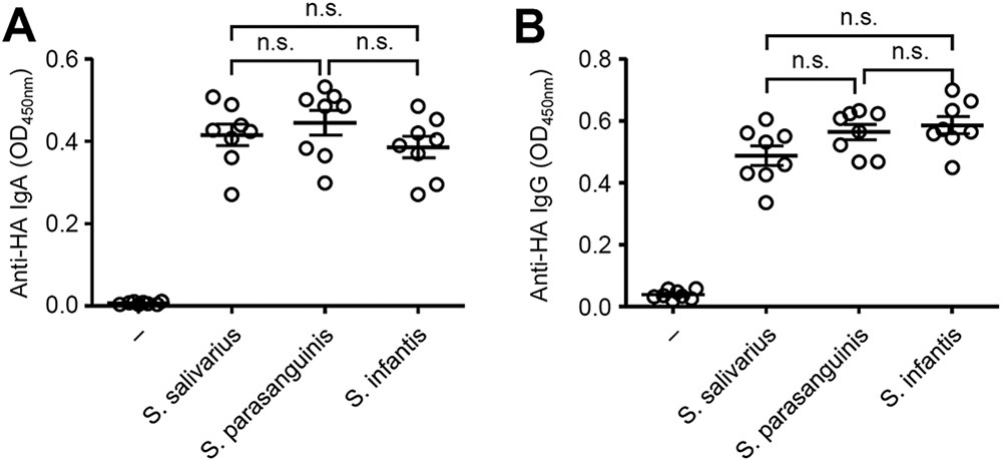
Adjuvant activity of *S. salivarius*, *S. parasanguinis*, and *S. infantis* for intranasal vaccine. (A and B) Mice were immunized intranasally with quadrivalent HA vaccine with or without *S. salivarius*, *S. parasanguinis*, or *S. infantis* twice in a 3-week interval. Two weeks later, the nasal washes and sera were collected and the HA-specific nasal IgA and serum IgG titers were determined by ELISA. Open circles indicate values for individual mice. The data are from two independent experiments (mean ± SEM). n.s., not significant (one-way ANOVA and Tukey’s test).

While the upper respiratory tract contains commensal bacteria (12, 13), we found that relative amounts of 16S rRNA and culturable bacteria in the nasal mucosal surface were significantly lower than those in the oral cavity (Fig. 3A to D). Thus, we next examined whether intranasal supplementation of oral bacteria enhances nasal IgA response to intranasally administered antigens. Notably, intranasal vaccination with HA and cultured oral bacteria from mice or a healthy volunteer significantly enhanced the HA-specific nasal IgA and serum IgG responses (Fig. 3E and F). In addition, the oral bacteria from a healthy volunteer stimulated the HA-specific nasal IgA and serum IgG responses in a dose-dependent manner (Fig. 3G and H). Next, we compared the ability of isolated bacterial strains from an oral wash sample of a healthy volunteer to stimulate the HA-specific antibody response. To this end, we immunized mice intranasally with HA and Streptococcus salivarius, Streptococcus parasanguinis, or Streptococcus infantis. Mice immunized with HA and each isolated bacterial strain induced comparable levels of the HA-specific nasal IgA and serum IgG responses (Fig. 4), suggesting that a specific strain of the oral bacteria is unlikely to account for the adjuvant activity.

### Myd88-dependent signaling in the hematopoietic compartment is required for adjuvant activity of intranasally administered oral bacteria.

**FIG 5:**
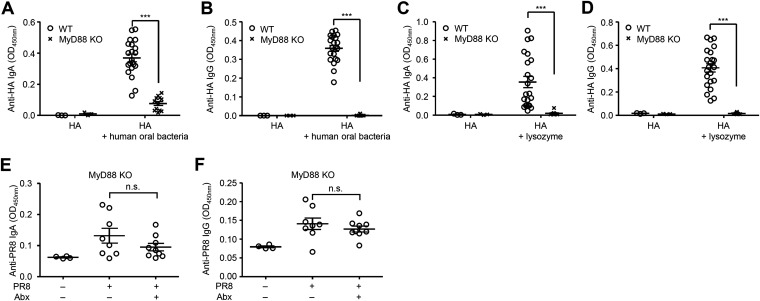
Disruption of nasal bacteria or intranasal administration of cultured oral bacteria induces the HA-specific antibody responses in a MyD88-dependent manner. (A to D) WT and MyD88-deficient mice were immunized intranasally with a quadrivalent HA vaccine with or without cultured oral bacteria from a healthy volunteer (A and B) or lysozyme (C and D) twice in a 3-week interval. Two weeks later, the nasal washes and sera were collected and the HA-specific nasal IgA and serum IgG titers were determined by ELISA. (E and F) MyD88-deficient mice were inoculated intranasally with an antibiotic cocktail (Abx) for 5 consecutive days. Two days later, mice were intranasally infected with 1,000 PFU of A/PR8 virus. The nasal washes and sera were collected at 4 weeks p.i., and the virus-specific nasal IgA and serum IgG titers were determined by ELISA. Open circles indicate values for individual mice. The data are from two independent experiments (mean ± SEM). ***, *P < *0.001; n.s., not significant (one-way ANOVA and Tukey’s test).

**FIG 6:**
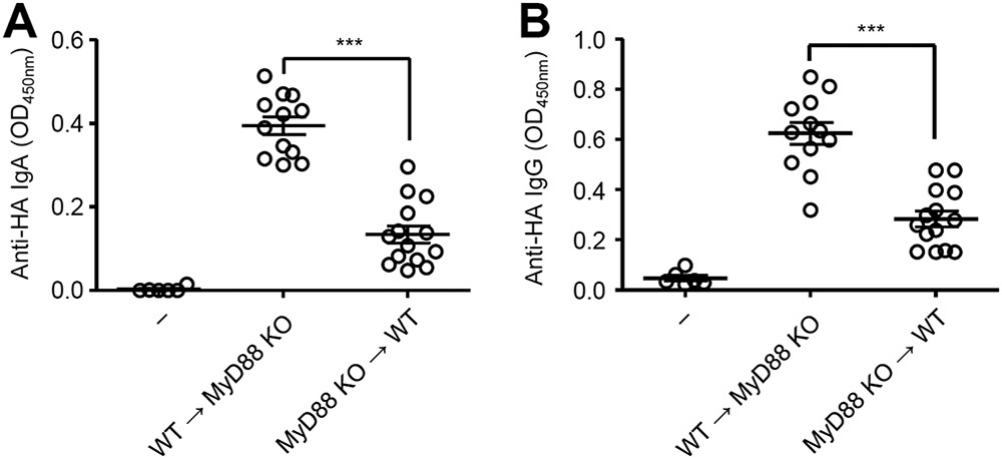
Myd88-dependent signaling in the hematopoietic compartment was required for adjuvant activity of intranasally administered oral bacteria. (A and B) WT→MyD88 KO and MyD88 KO→WT BM chimeric mice were immunized intranasally with quadrivalent HA vaccine with or without cultured oral bacteria from a healthy volunteer twice in a 3-week interval. Two weeks later, the nasal washes and sera were collected, and the HA-specific nasal IgA and serum IgG titers were determined by ELISA. Open circles indicate values for individual mice. The data are from two independent experiments (mean ± SEM). ***, *P < *0.001 (one-way ANOVA and Tukey’s test).

Next, we wished to determine the innate immune signaling through pattern-recognition receptors (PRRs) required for adjuvant activity of the oral bacteria. To this end, we immunized wild-type (WT) and MyD88-deficient mice intranasally with HA, cultured oral bacteria from a healthy volunteer, and measured the HA-specific nasal IgA and serum IgG responses. The HA-specific nasal IgA and serum IgG responses were found to be completely dependent on MyD88 (Fig. 5A and B). In addition, lysozyme-induced disruption of nasal bacteria stimulated the HA-specific nasal IgA and serum IgG responses in a MyD88-dependent manner (Fig. 5C and D). Furthermore, depletion of commensal bacteria in the upper respiratory tract did not enhance the virus-specific nasal IgA and serum IgG levels in MyD88-deficient mice following influenza virus infection (Fig. 5E and F). These data suggested that commensal bacteria in the upper respiratory tract are unlikely to inhibit influenza virus-specific antibody responses and highlighted the possibility that intranasal application of antibiotics could release bacterial PAMPs that act as mucosal adjuvants for influenza virus-specific antibody responses via the MyD88 signaling pathway. To determine the cellular compartment responsible for the adjuvant activity of oral bacteria, we generated bone marrow (BM) chimeric mice in which only the hematopoietic (WT→MyD88^–/–^) or the stromal cells (MyD88^–/–^→WT) expressed MyD88. After intranasal vaccination with HA and oral bacteria, the HA-specific nasal IgA and serum IgG responses were significantly reduced in MyD88^–/–^→WT BM chimeric mice compared with those in WT→MyD88^–/–^ BM chimeric mice (Fig. 6). These data indicate that MyD88-dependent signaling in the hematopoietic, but not stromal, compartment is required for adjuvant activity of intranasally administered oral bacteria.

### Oral bacteria combined with an intranasal vaccine protect from influenza virus and SARS-CoV-2 infection.

**FIG 7:**
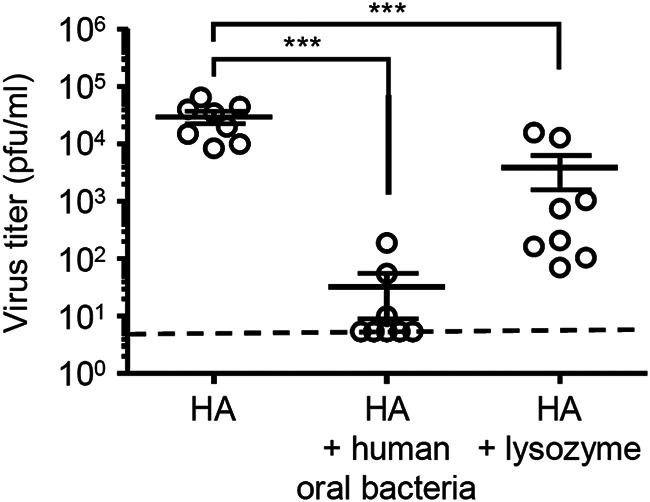
Protective effects of oral bacteria-adjuvanted intranasal vaccine against influenza virus infection. Mice were immunized intranasally with a quadrivalent HA vaccine with or without cultured oral bacteria from a healthy volunteer or lysozyme twice in a 3-week interval. Two weeks after the last vaccination, mice were challenged with 1,000 PFU of A/Narita/1/09 (pdm09). The nasal wash of influenza virus-infected mice was collected at 3 days postinfection, and viral titers were determined by plaque assay. Open circles indicate values for individual mice. The dashed line indicates the limit of virus detection. The data are from two independent experiments (mean ± SEM). ***, *P < *0.001 (one-way ANOVA and Tukey’s test).

**FIG 8:**
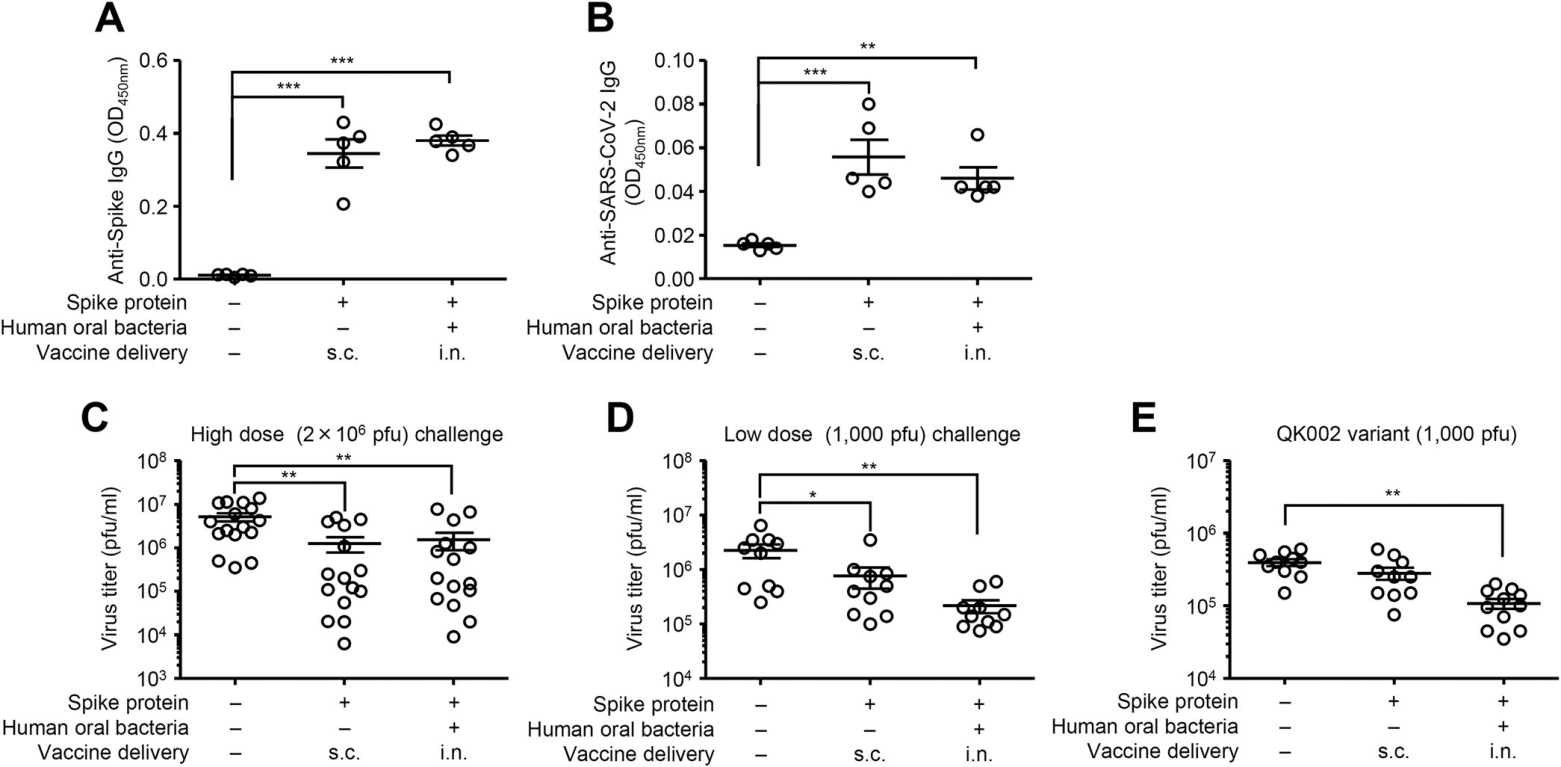
Protective effects of oral bacteria-adjuvanted intranasal vaccine against SARS-CoV-2 infection. (A to E) Hamsters were immunized intranasally with the spike protein of SARS-CoV-2 with cultured oral bacteria from a healthy volunteer twice in a 3-week interval. As controls, hamsters were left unimmunized or subcutaneously immunized with the spike protein alone twice in a 3-week interval. Two weeks after the last vaccination, hamsters were challenged with 2 × 10^6^ (A to C) or 1,000 PFU (D and E) of SARS-CoV-2. (A and B) Sera were collected at 3 days postinfection. The recombinant spike protein- (A) or formalin-inactivated SARS-CoV-2 virion- (B) specific serum IgG antibody titers were determined by ELISA. (C to E) Two weeks after the last vaccination, hamsters were challenged with 2 × 10^6^ (C) or 1,000 PFU (D and E) of SARS-CoV-2 (C and D) or its QK002 variant (E). The lung wash of SARS-CoV-2-infected hamsters was collected at 3 days postinfection, and viral titers were determined by plaque assay. Open circles indicate values for individual hamsters. The data are from two or three independent experiments (mean ± SEM). *, *P < *0.05; **, *P < *0.01; ***, *P < *0.001 (one-way ANOVA and Tukey’s test).

Finally, we examined the protective effects of intranasal vaccination with oral bacteria-adjuvanted vaccine against influenza virus and SARS-CoV-2 infection. To this end, we immunized mice intranasally with a quadrivalent influenza HA vaccine containing A/California/7/2009 HA together with cultured oral bacteria or lysozyme. Two weeks after the second vaccination, we challenged vaccinated mice intranasally with a heterologous A/Narita/1/2009 (pdm09) strain (Fig. 7). Mice immunized with the HA vaccine adjuvanted with oral bacteria or lysozyme significantly reduced the virus titer compared with control mice immunized with the HA vaccine alone (Fig. 7). We next assessed the protective effects of intranasal vaccination with oral bacteria-adjuvanted SARS-CoV-2 spike protein against SARS-CoV-2 infection in Syrian hamsters. To this end, we immunized hamsters intranasally with a recombinant SARS-CoV-2 spike protein and cultured oral bacteria from a healthy volunteer. We immunized hamsters subcutaneously with the spike protein alone as a control. Both the spike- and the virion-specific serum IgG levels were significantly elevated in immunized hamsters (Fig. 8A and B). These immunized hamsters significantly reduced the virus titer compared with naive animals following high- (2 × 10^6^ PFU) and low-dose (1,000 PFU) challenge (Fig. 8C and D). In addition, intranasally but not subcutaneously immunized hamsters significantly reduced the virus titer compared with naive animals following a low dose (1,000 PFU) of a SARS-CoV-2 QK002 variant (lineage B.1.1.7) (Fig. 8E). These data collectively indicated that oral bacteria combined with an intranasal vaccine induce protective antibody responses to influenza virus and SARS-CoV-2 infection (see Fig. S1 in the supplemental material).

10.1128/mBio.01598-21.1 FIG S1 Proposed mechanism by which intranasal supplementation of cultured oral bacteria or disruption of nasal bacteria enhance antibody responses to intranasally administered vaccines. While the upper respiratory tract contains commensal bacteria, the relative amounts of 16S rRNA and culturable bacteria in nasal mucosal surface are significantly lower than those in the oral cavity. Thus, intranasal administration of influenza virus hemagglutinin (HA) or the SARS-CoV-2 spike (S) protein alone seems to be insufficient for inducing the vaccine-specific antibody responses. However, intranasal supplementation of cultured oral bacteria or disruption of nasal bacteria stimulates antibody responses to intranasally administered vaccines in a MyD88-dependent manner. Download FIG S1, TIF file, 1.6 MB. Copyright © 2021 Nagai et al. 2021 Nagai et al. https://creativecommons.org/licenses/by/4.0/ This content is distributed under the terms of the Creative Commons Attribution 4.0 International license.

## DISCUSSION

The innate immune system, the first line of defense against pathogens, uses PRRs to detect PAMPs. The recognition of influenza virus by PRRs plays a key role not only in limiting virus replication at early stages of infection but also in initiating the virus-specific adaptive immune responses. In addition, previous studies have demonstrated that gut commensal microbiota play a key role in innate and adaptive immune defense against influenza virus infection (15–17, 19, 21, 22). Furthermore, recent studies have indicated the roles of nasal bacteria in innate antiviral resistance to influenza virus infection or severity of the diseases (24, 25). However, it remains unclear whether nasal bacteria critically regulate the generation of influenza virus-specific adaptive immune responses after influenza virus infection. In this study, we demonstrated that intranasal administration of antibiotics before influenza virus infection enhanced the virus-specific antibody response in a MyD88-dependent manner. Surprisingly, depletion of nasal bacteria by intranasal administration of antibiotics before influenza virus infection significantly reduced the virus titer at 2 days postinfection. This finding is consistent with a previous report showing that antibiotic treatment significantly reduce influenza virus replication at 6 hours postinfection (23). Intranasal application of antibiotics suppressed influenza virus replication through at least two possible mechanisms. First, intranasal administration of antibiotics enhances host resistance to influenza virus infection in a microbiota-independent manner (23). Second, disruption of nasal bacteria by intranasal antibiotic treatment may release PAMPs from the antibiotic-killed bacteria, which stimulate innate antiviral immune responses to suppress influenza virus replication (26). After 3 and 5 days postinfection, the viral replication in the upper respiratory tract became comparable between antibiotic-treated and control groups, indicating that the levels of influenza virus replication in the upper respiratory tract are unlikely to account for increased levels of the virus-specific antibody responses in antibiotic-treated mice.

Since the primary targets of influenza virus are the nasal epithelial cells in the upper respiratory tract, it is beneficial to induce the virus-specific nasal IgA antibody at the nasal mucosal epithelium. However, intranasal vaccination with a split-virus vaccine alone is often insufficient to elicit proper immune responses at the upper respiratory tract. Therefore, adjuvants are required for a given vaccine to induce the vaccine-specific nasal IgA response. In developing intranasal vaccines, cholera toxin (CT) and Escherichia coli heat-labile toxin (LT) have been used as adjuvants to enhance the nasal immune response (27). Although CT and LT are effective adjuvants for enhancing mucosal immune responses, including secretory IgA responses, they have some side effects in humans, including Bell’s palsy and nasal discharge (28). Therefore, several adjuvants that are as effective as CT or LT and are also safe for human use have been developed for clinical application with an intranasal influenza vaccine (6–11). In this study, we show that intranasal vaccination with influenza virus HA vaccine and cultured oral bacteria from a healthy human volunteer induced significant levels of the vaccine-specific nasal IgA and serum IgG responses in a dose-dependent manner. All commensal bacterial strains tested, including *S. salivarius*, *S. parasanguinis*, or *S. infantis*, induced comparable levels of the HA-specific nasal IgA and serum IgG responses, suggesting that the adjuvant activity of the oral bacteria is not strain specific. In addition to cultured oral bacteria from a healthy human volunteer, we demonstrated that disruption of nasal bacteria by lysozyme induced significant levels of the vaccine-specific antibody responses. Although relative amounts of nasal bacteria were significantly lower than those in the oral cavity, disruption of nasal bacteria by lysozyme could stimulate the vaccine-specific antibody responses. In mice, nasal commensal microbiota are predominantly composed of Gram-positive bacteria, including *Lactobacillus* spp., *Bacillus* spp., Staphylococcus spp., and Streptococcus spp. (15). In addition, *Lactobacillus* spp. were found to contain larger amounts of double-stranded RNA than the pathogenic bacteria (29). Since activation of TLRs by different PAMPs, such as poly(I:C) and zymosan, synergistically enhanced the nasal IgA response to an intranasally administered influenza virus HA vaccine (8), disruption of nasal bacteria could stimulate different TLRs to enhance the vaccine-specific antibody responses (Fig. S1). Most TLRs signal through the adaptor protein MyD88 (30, 31). Although nasal epithelial cells express various TLRs (32, 33), deficiency of MyD88 in the stromal compartment did not significantly affect the levels of nasal IgA and serum IgG responses following intranasal vaccination with influenza virus HA and cultured oral bacteria. Instead, MyD88-dependent signaling in the hematopoietic cells was required for the adjuvant activity of intranasally administered oral bacteria. These data are consistent with previous studies showing that both TLR-induced dendritic cell maturation and B-cell activation are required for optimal antibody responses to T-dependent antigens (34, 35).

In summary, our study demonstrated the effects of commensal microbiota in the upper respiratory tract in the induction of the virus-specific adaptive immune responses after influenza virus infection or intranasal vaccination. Our data indicated that disruption of nasal bacteria by lysozyme or intranasal supplementation of oral bacteria from a healthy volunteer enhanced nasal IgA and serum IgG antibody responses to intranasally administered influenza virus HA or SARS-CoV-2 S proteins (Fig. S1). The vaccinated animals significantly reduced the virus titer compared with naive animals following SARS-CoV-2 challenge, but the protective efficacy of intranasal vaccination of hamsters with oral bacteria combined with a subunit vaccine was limited compared with that DNA or inactivated whole-virus vaccines (36, 37). Although we detected similar levels of the SARS-CoV-2-specific serum IgG antibodies in immunized hamsters, we observed a reduced viral burden in the lung of the intranasally immunized group, suggesting that the virus-specific mucosal IgA antibodies play an important role in limiting the virus replication (38). However, we were unable to measure the virus-specific IgA responses in immunized hamsters because of a lack of an anti-hamster IgA antibody. Thus, further studies are needed to establish the safety and efficacy of this vaccination method in hamsters and an additional animal model, such as nonhuman primate.

## MATERIALS AND METHODS

### Mice.

Age- and sex-matched BALB/c mice obtained from Japan SLC, Inc., were used as WT controls. MyD88-deficient BALB/c mice were purchased from Oriental Bioservice (Kyoto, Japan). All animal experiments were performed in accordance with the University of Tokyo’s Regulations for Animal Care and Use, which were approved by the Animal Experiment Committee of the Institute of Medical Science, the University of Tokyo (approval number PA17-69).

### Cells.

Madin-Darby canine kidney (MDCK) cells were grown in Eagle’s minimal essential medium (E-MEM; Nacalai Tesque) supplemented with 10% fetal bovine serum (FBS), penicillin (100 U/ml), and streptomycin (100 μg/ml). VeroE6 cells stably expressing transmembrane protease serine 2 (VeroE6/TMPRSS2; JCRB Cell Bank 1819) were maintained in Dulbecco’s modified Eagle’s medium (DMEM) low glucose (catalog [cat] number 08456-65; Nacalai Tesque) supplemented with 10% FBS, penicillin (100 U/ml), streptomycin (100 μg/ml), and G418 (1 mg/ml) (39).

### Depletion of nasal bacteria *in vivo*.

The antibiotic cocktail consisted of ampicillin sodium salt (1 g/liter), neomycin sulfate (1 g/liter), metronidazole (1 g/liter), vancomycin hydrochloride (0.5 g/liter), gentamicin (10 mg/liter), penicillin (100 U/ml), streptomycin (100 U/ml), and amphotericin B (0.25 mg/liter) (40). For intranasal treatment, mice were anesthetized and 5 μl of antibiotic was administered dropwise into each nostril using a pipette tip. All antibiotics with the exception of vancomycin hydrochloride were obtained from Nacalai Tesque. Vancomycin hydrochloride was obtained from Duchefa Biochemie.

### Virus infection.

WT A/Puerto Rico/8/34 (A/PR8) and A/Narita/1/09 (pdm09) influenza viruses were grown in allantoic cavities of 10-day-old fertile chicken eggs at 35°C for 2 days (41). Viral titer was quantified by a standard plaque assay using MDCK cells, and the viral stock was stored at −80°C (42). For intranasal infection, mice were fully anesthetized by intraperitoneal (i.p.) injection of a pentobarbital sodium (Somnopentyl; Kyoritsu Seiyaku Co., Ltd., Tokyo, Japan) and then infected by intranasal application of 30 μl of virus suspension (1,000 PFU of A/PR8 or pdm09 in phosphate-buffered saline (PBS)). This procedure leads to an upper and lower respiratory tract infection (40).

SARS-CoV-2/UT-NCGM02/Human/2020/Tokyo (43) and a QK002 variant (lineage B.1.1.7) were amplified on VeroE6/TMPRSS2 cells and stored at −80°C until use. The infectious titer was determined by a standard plaque assay using VeroE6/TMPRSS2 cells, as described previously (43). For intranasal infection, 1-month-old female Syrian hamsters (Japan SLC Inc.) were fully anesthetized by i.p. injection of a pentobarbital sodium (Somnopentyl; Kyoritsu Seiyaku Co., Ltd., Tokyo, Japan) and then infected intranasally with 2 × 10^6^ or 1,000 PFU (in 100 μl) of SARS-CoV-2. All experiments with SARS-CoV-2 were performed in enhanced biosafety level 3 (BSL3) containment laboratories at the University of Tokyo, in accordance with the institutional biosafety operating procedures.

### Vaccination.

For intranasal infection, mice were fully anesthetized by i.p. injection of pentobarbital sodium (Somnopentyl; Kyoritsu Seiyaku Co., Ltd., Tokyo, Japan) and then infected intranasally by dropping 2 μl of PBS containing 1,000 PFU of A/PR8 into the nostril. The quadrivalent inactivated influenza vaccine (split-product virus vaccines and hemagglutinin [HA] vaccine) prepared for the 2015 to 2016 season and including A/California/7/2009 (H1N1), A/Switzerland/9715293/2013 (H3N2), B/Phuket/3073/2013, and B/Texas/2/2013 were purchased from Kaketsuken (Kumamoto, Japan). Mice were immunized by intranasal administration of the quadrivalent HA vaccine containing 150 ng of each HA with or without 5 μg of lipopolysaccharide (LPS; InvivoGen), 5 μg of poly(I:C) (InvivoGen), 250 μg of lysozyme (Thermo Fisher Scientific), or 1 mg of cultured oral bacteria from a healthy volunteer.

A SARS-CoV-2 spike S1+S2 ECD-His recombinant protein was purchased from Sino Biological Inc. (cat number 40589-V08B1). Hamsters were immunized intranasally with 3 μg of the recombinant spike protein with 1 mg of cultured oral bacteria from a healthy volunteer. We immunized hamsters subcutaneously with 3 μg of the spike protein alone as a control.

### Clinical specimens.

Oral and nasal washes were collected from a healthy volunteer by rinsing the mouth with 50 ml of saline or washing the nasal cavity with 50 ml of saline using a syringe. The research protocol was approved by the Research Ethics Review Committee of the Institute of Medical Science, the University of Tokyo (approval number 2019-42-1121). For preparation of the oral bacterial adjuvant, oral wash samples were grown in brain heart infusion broth (BD 237500) at 37°C overnight, washed repeatedly, and resuspended in PBS (200 μg/ml).

### Bacterial recovery and identification.

Oral and nasal washes were collected from a healthy volunteer as described above. Aliquots of 100 μl of serial 10-fold dilutions of the oral and nasal washes were inoculated into brain heart infusion agar plates (BD 252109). After incubation at 37°C overnight under the aerobic conditions, the bacterial colonies were grown in brain heart infusion broth (BD 237500) at 37°C overnight. Bacterial DNA was isolated as described previously (40). A 300-bp portion of the 16S rRNA was amplified by PCR using specific primer pairs of 515F (5′-GTGCCAGCMGCCGCGGTAA-3′) and 806R (5′-GGACTACHVGGGTWTCTAAT-3′), purified (Qiagen), and sequenced, and the sequence was compared by BLAST analysis to known bacterial sequences.

### Bone marrow chimera.

Bone marrow chimeras were generated as described (44). WT and MyD88-deficient mice were γ-irradiated with 6 Gy, then were reconstituted with 5 × 10^6^ bone marrow cells of the appropriate genotype via intravenous (i.v.) injection, and allowed to recover for 8 weeks before vaccination.

### Measurement of virus titers.

For measurement of influenza virus titer, bronchoalveolar (BAL) fluid was collected by washing the trachea and lungs of mice twice by injecting a total of 2 ml PBS containing 0.1% bovine serum albumin (BSA). The virus titer was measured as follows: aliquots of 200 μl of serial 10-fold dilutions of the BAL fluid by PBS containing 0.1% BSA were inoculated into MDCK cells in 6-well plates. After 1 h of incubation, cells were washed with PBS thoroughly and overlaid with 2 ml of agar medium.

For measurement of SARS-CoV-2 titer, BAL fluid was collected by washing the trachea and lungs of hamsters twice by injecting a total of 2 ml DMEM containing 5% FBS. The virus titer was measured as follows: aliquots of 200 μl of serial 10-fold dilutions of the BAL fluid by DMEM containing 5% FBS were inoculated into VeroE6/TMPRSS2 cells in 6-well plates. After 1 h of incubation, cells were washed with PBS thoroughly and overlaid with 2 ml of agar medium. The number of plaques in each well was counted 2 days after inoculation.

### Enzyme-linked immunosorbent assay (ELISA).

Serum and nasal washes were collected from the immunized mice for measurement of the PR8- or HA-specific nasal IgA and serum IgG antibodies. Nasal washes were collected by washing the nasopharynx three times by injecting a total of 1 ml PBS containing 0.1% BSA. The levels of the PR8- or HA-specific nasal IgA and serum IgG antibodies were determined by ELISA as described previously (40, 45). In brief, a 96-well plate (cat number 442404; Nunc Maxisorp) was coated with formalin-inactivated PR8 virion or quadrivalent HA vaccine with carbonate buffer. After overnight incubation at 4°C, the coating antigen was removed and 100 μl per well of 2% FBS in PBS was added to the plates at room temperature for 1 h as a blocking solution. Serum samples were diluted 1:100 with 2% FBS in PBS. The blocking solution was removed and 100 μl of diluted serum samples or undiluted nasal wash samples were then plated in the wells and incubated for 2 h at room temperature. After wells were washed in PBS with 0.05% Tween 20, horseradish peroxidase (HRP)-conjugated goat anti-mouse IgG (1:2,000; cat number 115-035-003; Jackson Immunoresearch Laboratories) or goat anti-mouse IgA (1:2,000; cat number 626720; Invitrogen) antibodies were added to the wells for 1 h, and then the wells were washed and a 3,3′,5,5′-tetramethylbenzidine (TMB) solution (eBioscience) was added. Reactions were stopped with 1 M H_3_PO_4_, and absorbance was measured at 450 nm.

The levels of IgG antibodies against the SARS-CoV-2 spike protein or whole virus were detected by ELISA. In brief, a 96-well plate (cat number 442404; Nunc Maxisorp) was coated with a recombinant SARS-CoV-2 spike protein (Sino Biological Inc.) or formalin-inactivated SARS-CoV-2 virion with carbonate buffer. After overnight incubation at 4°C, the coating antigen was removed and 100 μl per well of 2% FBS in PBS was added to the plates at room temperature for 1 h as a blocking solution. Serum samples were diluted 1:100 with 2% FBS in PBS. The blocking solution was removed, and 100 μl of diluted serum samples were then plated in the wells and incubated for 2 h at room temperature. After being washed in PBS with 0.05% Tween 20, wells received an HRP-conjugated goat anti-Syrian hamster IgG (1:10,000; cat number ab6892; abcam) antibody for 1 h, and then wells were washed and a TMB solution (eBioscience) was added. Reactions were stopped with 1 M H_3_PO_4_, and absorbance was measured at 450 nm.

### Quantification and statistical analysis.

Statistical significance was tested by one-way analysis of variance (ANOVA) followed by Tukey’s test or unpaired *t* tests with PRISM software (version 5; GraphPad software). Data are presented as mean ± SEM. Statistical details can be found directly in the figure legends. *P* values of less than 0.05 were considered statistically significant.
