# Supplementary figures and images for: Oral Bacteria Combined with an Intranasal Vaccine Protect from Influenza A Virus and SARS-CoV-2 Infection
Source: mBio. 2021 Aug 17;12(4):e01598-21. doi: 10.1128/mBio.01598-21 (PMC8406166; doi:10.1128/mBio.01598-21)

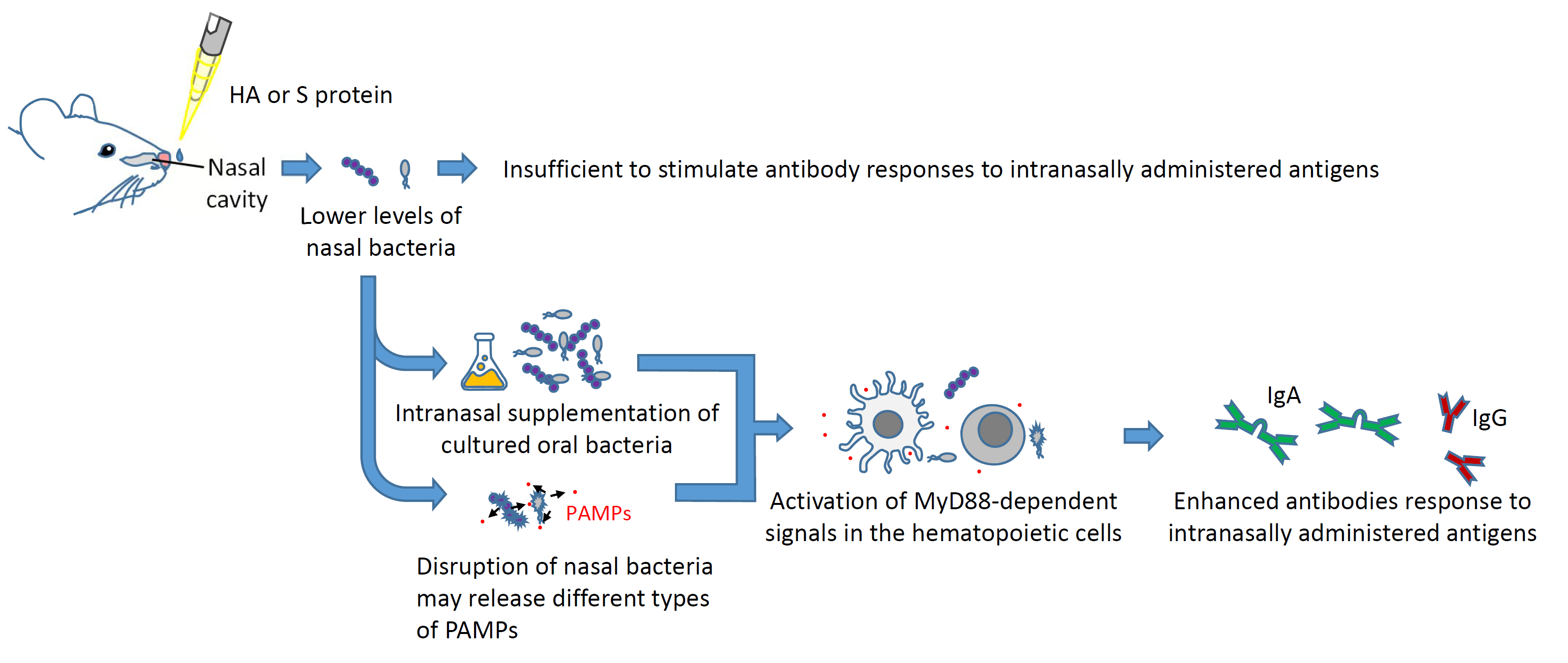

Supplement: FIG S1 [file mbio.01598-21-sf001.tif]
